# Long non-coding RNA HOTAIR induces GLI2 expression through Notch signalling in systemic sclerosis dermal fibroblasts

**DOI:** 10.1186/s13075-020-02376-9

**Published:** 2020-12-10

**Authors:** Christopher W. Wasson, Rebecca L. Ross, Rebecca Wells, Clarissa Corinaldesi, Ioanna Ch. Georgiou, Natalia A. Riobo-Del Galdo, Francesco Del Galdo

**Affiliations:** 1 Leeds Institute of Rheumatic and Musculoskeletal Medicine, Faculty of Medicine and Health, University of Leeds, Leeds, UK; 2 School of Molecular and Cellular Biology, Faculty of Biological Sciences, University of Leeds, Leeds, UK; 3 Leeds Institute of Medical Research, Faculty of Medicine and Health, University of Leeds, Leeds, UK; 4 Scleroderma Programme, NIHR Leeds Musculoskeletal Biomedical Research Centre, Leeds, UK

**Keywords:** Systemic sclerosis, Hedgehog signalling, Epigenetics, Long non-coding RNA, HOTAIR

## Abstract

**Objectives:**

Systemic sclerosis (SSc) is characterised by tissue fibrosis of the major organs of the body including the skin, lungs and heart. We have previously reported that the lncRNA HOTAIR plays a central role in the activation of SSc myofibroblasts, the key cellular elements of fibrosis. HOTAIR induces fibroblast activation through H3K27me3-mediated activation of the Notch signalling pathway. Here we aimed to identify the signalling events downstream of Notch that drive SSc myofibroblast activation.

**Methods:**

Patient fibroblasts were obtained from full-thickness forearm skin biopsies of 3 adult patients with SSc of recent onset. The lncRNA HOTAIR was expressed in healthy dermal fibroblasts by lentiviral transduction. Hedgehog signalling pathway was inhibited with GANT61 and GLI2 siRNA. Gamma secretase inhibitors RO4929097 and DAPT were used to block Notch signalling. GSK126 was used to inhibit Enhancer of Zeste 2 (EZH2).

**Results:**

Overexpression of HOTAIR in dermal fibroblasts induced the expression of the Hedgehog pathway transcription factor GLI2. This is mediated by activation of Notch signalling following epigenetic downregulation of miRNA-34a expression. Inhibition of H3K27 methylation and Notch signalling reduced expression of GLI2 in HOTAIR-expressing fibroblasts as well as in SSc dermal fibroblasts. Importantly, the inhibition of GLI2 function using GANT61 or siRNA mitigates the pro-fibrotic phenotype induced by HOTAIR.

**Conclusions:**

Our data indicates that GLI2 expression is stably upregulated in SSc myofibroblasts through HOTAIR and that GLI2 mediates the expression of pro-fibrotic markers downstream of Notch.

**Supplementary Information:**

The online version contains supplementary material available at 10.1186/s13075-020-02376-9.

## Introduction

Systemic sclerosis (SSc) is an autoimmune condition that initially presents in the skin of the patient’s hands and feet, where there is a build-up of extracellular matrix resulting in skin fibrosis. The disease progresses to the forearms and legs and in the most severe cases (diffuse SSc) to the trunk. Beyond skin fibrosis, SSc can cause tissue fibrosis in the internal organs (lungs, heart and kidneys). The key role of tissue fibroblasts in the disease has been well-characterised. A number of signalling pathways such as Transforming Growth Factor beta (TGF-β) [1–3], Sonic Hedgehog (Shh) [4–6] and Notch signalling [7, 8] have been implicated in driving myofibroblast activation, but their precise role and interplay is not fully understood. Epigenetic factors are known to play a key role in the ability of SSc myofibroblasts to maintain their phenotype when explanted from tissue [9, 10]. We have recently shown that the long non-coding RNA HOTAIR, in cooperation with the polycomb repressor complex 2 (PRC2), drives H3K27me3 histone methylation and silences miRNA-34a expression. This leads to increased expression of the miRNA-34a-target Notch1 which results in increasing Notch signalling and myofibroblast activation [11].

Notch signalling is an important regulator of cell to cell communication [12]. Upon ligand binding, the Notch receptor is cleaved by gamma secretases, which leads to the release of the Notch intracellular domain (NID). In turn, NID binds to the transcription factor CSL [13] and initiates transcription of a number of downstream transcription factors including Hes1 [14], which has previously been shown to be upregulated in SSc patient skin and fibroblasts [7, 11]. Inhibition of Notch signalling blocks the expression of pro-fibrotic markers in SSc fibroblasts [7, 11]. The downstream transcription factors that play a role in Notch-mediated SSc fibroblast activation remain unclear. Here we set out to investigate the molecular mechanism by which Notch promotes the pro-fibrotic phenotype of SSc fibroblasts.

Ringuette et al. have shown that activation of Notch in Müller glia induces GLI2 expression [15, 16]. These findings are consistent with the data showing that inhibition of Notch signalling in amyotrophic lateral sclerosis leads to reduced GLI2 levels [17]. GLI2 is the main transcriptional activator in canonical Hedgehog (Hh) signalling [18]. We and others have shown that GLI1 and GLI2 are upregulated in SSc skin and fibroblasts [5] and SHH pathway activation plays an important role in the pathogenesis of tissue fibrosis both in scleroderma and sclerotic graft versus host disease (GVHD) [5, 6]. These two lines of evidence led us to hypothesise that the activation of GLI2 observed during skin fibrosis could be at least in part driven by HOTAIR-induced NOTCH activation.

The Hh pathway is essential for normal embryonic development and plays a critical role in adult tissue maintenance [19]. Hh family members, such as SHH, initiate signalling through binding to the receptor Patched 1 (PTCH1) [20]. Receptor binding results in derepression of Smoothened (SMO) [21], a GPCR that mediates activation of downstream GLI transcription factors. GLI2 and GLI3 are constitutively expressed and kept inactive by phosphorylation-dependent proteasomal processing into transcriptional repressors [22, 23]. Activation of SMO prevents GLI2 and GLI3 processing and induces accumulation of full-length activators, which initiate transcription of target genes, among which are GLI1 and PTCH1 [21].

In this study, we show that HOTAIR expression is sufficient to drive Notch-dependent increase of GLI2 expression through Enhancer of Zeste Homology 2 (EZH2)-dependent repression of miRNA-34a and that inhibition of GLI2 is sufficient to reduce the pro-fibrotic phenotype of dermal fibroblasts.

## Materials and methods

### Patient cell lines

Full-thickness skin biopsies were surgically obtained from the forearms of 3 adult patients with SSc of recent onset, which was defined as disease duration of less than 18 months from the appearance of clinically detectable skin induration. The patients satisfied the 2013 ACR/EULAR criteria for the classification of SSc and had the diffuse cutaneous clinical subset as defined by LeRoy et al. [24]. Healthy and SSc patient fibroblasts were isolated and established from the biopsies as described [3]. Briefly, isolated healthy and SSc fibroblasts were retrovirally transduced with HTERT to immortalise the fibroblasts. Experiments were performed when the fibroblasts were between passages 3 and 6. All participants provided written informed consent to participate in this study. Informed consent procedure was approved by NRES-011NE to Dr. Francesco Del Galdo.

### Small molecule inhibitors

GSK126 is a EZH2 methylation transferase inhibitor. It was used at a final concentration of 5 μM and purchased from LKT Laboratories. The gamma secretase inhibitors R04929097 (Cayman Chemicals) and DAPT (Tocris) were used at final concentrations of 1 μM and 10 μM, respectively. The GLI inhibitor GANT61 was used at a final concentration of 10 μM (Selleck). All inhibitors were reconstituted in DMSO and added to fibroblasts in complete media for 48 h at 37 °C in a 5% CO_2_ atmosphere. The inhibitor concentrations were selected as they have been shown to efficiently block their respective pathways in SSc fibroblasts in previous studies [7, 8, 10, 11].

### Lentiviral transduction

Fibroblasts were grown from healthy control forearm biopsies and immortalised using retrovirus expressing human telomerase (hTERT) as previously outlined [3]. HOTAIR expression was then induced by transduction with GIPZ lentiviruses carrying HOTAIR gene sequence or scrambled RNA sequence as control in frame with puromycin resistance gene and GFP fluorochrome gene (Open Biosystems, Surrey, UK). For this purpose, cells were seeded at 50% confluence and infected with lentiviral particles in serum-free DMEM and incubated for 6 h, after which an additional 1 ml of DMEM containing 10% FCS was added and the cells were incubated for a further 72 h. Stably transduced cells were positively sorted for GFP fluorescence employing fluorescence-activated cell sorting in sterile conditions (BD INFLUX). Positively sorted cells were further selected in media containing 1.0 μg/ml puromycin (Life Technologies) for 10 days.

### miRNA-34a transfections

Fibroblasts were transfected with 67 nM of miRNA-34a mimic or a negative control scramble miRNA (both from Qiagen) using Lipofectamine 2000 transfection reagent. Fibroblasts were then incubated for 48 h prior to harvesting.

### GLI2 siRNA transfection

A pool of four siRNA duplexes specific for GLI2 (targeting different regions of the mRNA) or a negative control scramble siRNA (both from Qiagen) were transfected into fibroblasts using Lipofectamine 2000 transfection reagent. Fibroblasts were then incubated for 48 h prior to harvesting.

### Sonic hedgehog stimulation

Scramble and HOTAIR-expressing fibroblasts were serum starved for 24 h with DMEM containing 0.5% FBS, then stimulated with 2 μg/ml SHH ligand (R&D systems) for a further 24 h.

### Immunofluorescence

Fibroblasts were grown on glass coverslips, fixed with 4% paraformaldehyde and permeabilised with 0.1% Triton X-100 in PBS. Coverslips were stained with a mouse α-SMA antibody (Abcam ab7817) or acetylated α-tubulin and visualised with an Alexa 594-conjugated Ab (Thermo-Life Technologies). The nuclei were counterstained with 4′,6-diamidino-2-phenylindole (DAPI) and mounted in Prolong Gold (Invitrogen). Cilium lengths were measured using ImageJ software. Thirty cilia were measured for each experimental condition from 4 independent experiments.

### Western blotting

Total protein was extracted from fibroblasts in RIPA buffer and resolved by SDS-PAGE (10–15% Tris-Glycine), transferred onto Hybond nitrocellulose membrane (Amersham Biosciences) and probed with antibodies specific for alpha smooth muscle actin (α-SMA) (Abcam ab7817), Notch1 (Cell signalling 3608), H3K27me3 (Abcam ab6002), β-Actin (Sigma A5441), GLI1 (Santa Cruz sc-515751) and GLI2 (Novus Biologicals AF3635). Immunoblots were visualised with species-specific HRP-conjugated secondary antibodies (Sigma) and ECL (Thermo/Pierce) on a Biorad ChemiDoc imaging system.

### Quantitative real-time PCR

RNA was extracted from cells using the RNA extraction kit (Zymo Research) following the manufacturer's protocols. One microgramme of RNA was reverse transcribed using the cDNA synthesis kit (Thermo). Q-RT-PCR analysis was performed using the SYBR Green PCR mastermix kit (Thermo) using the PCR machine (Thermo) with primers specific for GLI1 (forward GGACCTGCAGACGGTTATCC, reverse AGCCTCCTGGAGATGTGCAT), GLI2 (forward TTTATGGGCATCCTCTCTGG, reverse TTTTGCATTCCTTCCTGTCC), Ptch1 (forward CGATGGAGTCCTTGCCTACAA, reverse CCACCAGACGCTGTTTAGTCA), collagen type 1A1 (forward CCTCCAGGGCTCCAACGAG, reverse TCTATCACTGTCTTGCCCCA), type 1A2 (forward GATGTTGAACTTGTTGCTGAGC, reverse TCTTTCCCCATTCATTTGTCTT), alpha SMA (forward TGTATGTGGCTATCCAGGCG, reverse AGAGTCCAGCACGATGCCAG), CTGF (forward GTGTGCACTGCCAAAGATGGT, reverse TTGGAAGGACTCACCGCT), Notch 1 (forward CCAGAACTGTGAGGAAAATATCG, reverse TCTTGCAGTTGTTTCCTGGAC), Hes1 (forward TACCCAGCCAGTGTCAAC, reverse CAGATGCTGTCTTTGGTTTATCC) and GAPDH (forward ACCCACTCCTCCACCTTTGA, reverse CTGTTGCTGTAGCCAAATTCGT). The data obtained was analysed according to the ΔΔ C_t_ method. GAPDH served as housekeeping gene.

### Statistical analysis

Results were presented as mean and standard error. Statistical analysis was performed using a two-tailed, paired Student’s *t* test.

## Results

### Overexpression of HOTAIR in dermal fibroblasts induces NOTCH-dependent GLI2 expression

**Fig. 1:**
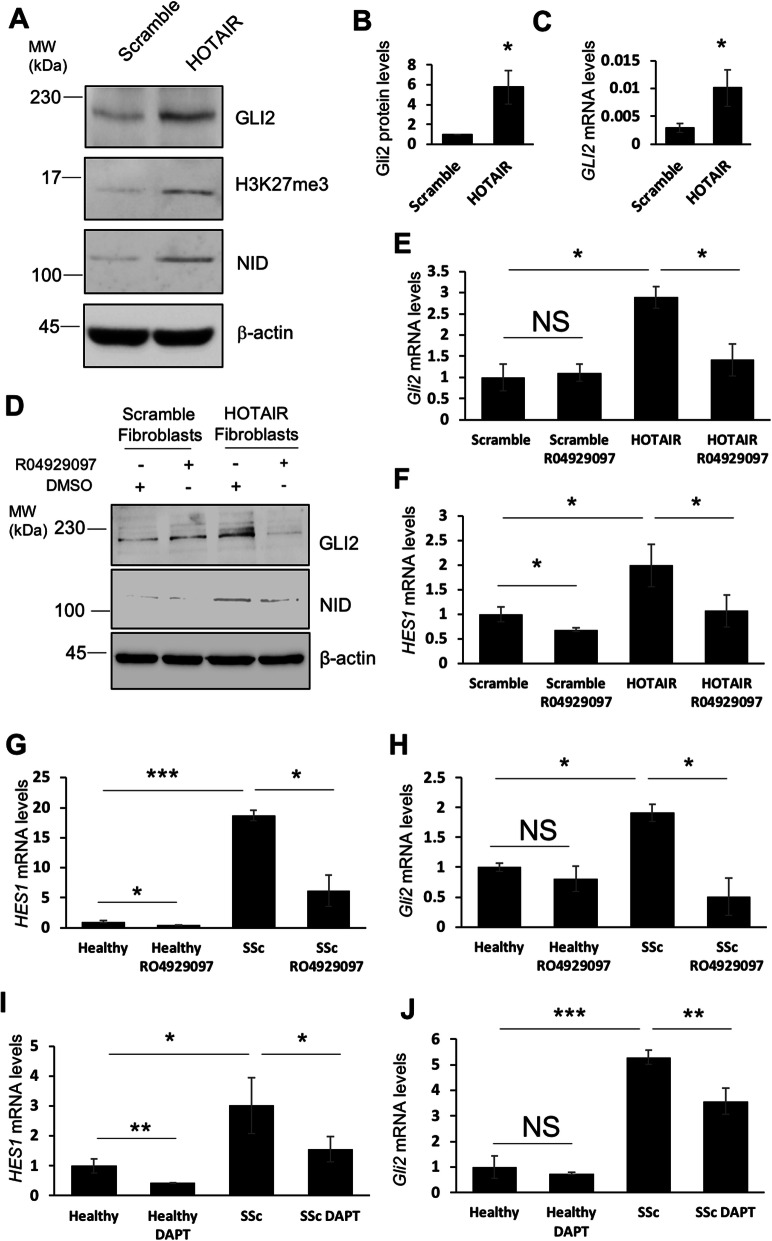
HOTAIR expression in dermal fibroblasts increases Notch-dependent GLI2 transcription. Dermal fibroblasts were transduced with lentiviruses containing a scramble or HOTAIR sequence. RNA and protein were extracted from these cells. **a** GLI2, H3K27m3 and NID protein levels were assessed by western blot. β-actin was used as a loading control. **b** Graph represents mean and standard error of densitometry analysis of GLI2 western blot. **c** *GLI2* transcript levels were analysed. Graphs represent the mean and standard error for three independent experiments. RNA and protein were extracted from scramble and HOTAIR-expressing fibroblasts. In addition, scramble and HOTAIR-expressing fibroblasts were treated with the gamma secretase inhibitor R04929097. **d** GLI2 and NID protein levels were assessed by western blot. β-actin was used as a loading control. *GLI2* (**e**) and *HES1* (**f**) transcript levels were analysed. Graphs represent the mean and standard error for three independent experiments. RNA was extracted from healthy and SSc fibroblasts. In addition, healthy and SSc fibroblasts were treated with the gamma secretase inhibitor R04929097. *HES1* (**g**) and *GLI2* (**h**) transcript levels were analysed. Graphs represent the mean and standard error for three independent experiments. RNA was extracted from healthy and SSc fibroblasts. In addition, healthy and SSc fibroblasts were treated with the gamma secretase inhibitor DAPT. *HES1* (**i**) and *GLI2* (**j**) transcript levels were analysed. Graphs represent the mean and standard error for three independent experiments.*p < 0.05, **p < 0.01, ***p < 0.001

Notch signalling is known to enhance expression of the transcription factor GLI2 [16, 17], and HOTAIR is known to regulate Notch expression in fibroblasts [11]. Therefore, we first investigated whether HOTAIR regulates the expression of GLI2 in dermal fibroblasts. Dermal fibroblasts overexpressing HOTAIR were generated as previously described [11] and showed stable H3K27me3 increased levels, as expected (Fig. 1a), since HOTAIR exerts a stimulatory function on the PRC2 complex [25]. As previously shown, HOTAIR induced an increase in NOTCH signalling as determined by increased levels of the transcriptionally active NOTCH intracellular domain (NID) (Fig. 1a). HOTAIR overexpression increased GLI2 expression at both protein (Fig. 1a, b) and mRNA levels (Fig. 1c) by 6- and 3-fold, respectively (*p* < 0.05 for both). To determine whether HOTAIR-mediated GLI2 upregulation was the consequence of enhanced Notch signalling, we investigated the levels of GLI2 in the presence of the gamma secretase inhibitor R04929097. As shown in Fig. 1d and e, R04929097 treatment (1 μM for 48 h) reduced GLI2 expression in HOTAIR-expressing fibroblasts but did not alter GLI2 levels in the scramble fibroblasts. We confirmed that R04929097 was effective in inhibiting NOTCH signalling, as we observed reduced levels of NID and reduced transcription of the *HES1* gene, a downstream target of Notch (Fig. 1d, f).

Importantly, SSc patient-derived fibroblasts showed an increase of *HES1* mRNA levels (Fig. 1g), consistent with previously reported data [11]. *HES1* increased expression was effectively suppressed by R04929097. In this setting, inhibition of NOTCH signalling reduced the levels of *GLI2* to a level undistinguishable from that of a healthy control (Fig. 1h), suggesting that upregulation of NOTCH signalling in SSc drives GLI2 upregulation. R04929097 did not alter *GLI2* levels in the healthy fibroblasts (Fig. 1h). To rule out off-target effects of the gamma secretase inhibitor, we used a second gamma secretase inhibitor (DAPT) with a different mode of action. Consistent with RO4929097, inhibiting Notch signalling in SSc fibroblasts with DAPT reduced *HES1* (Fig. 1i) and *GLI2* (Fig. 1j) transcript levels.

Interestingly, HOTAIR-induced GLI2 played no role in the maintenance of NOTCH1 expression in dermal fibroblasts, as we observed no reduction in NOTCH1 expression in HOTAIR-expressing fibroblasts transfected with GLI2 siRNA (Suppl. Fig. 1A-B) or treated with the GLI inhibitor GANT61 (Suppl. Fig. 1C). Similarly, depletion of GLI2 in SSc fibroblasts did not affect NOTCH1 levels (Suppl. Fig. 1D-E).

### Upregulation of GLI2 in HOTAIR-expressing fibroblasts is mediated by miRNA-34a suppression

**Fig. 2:**
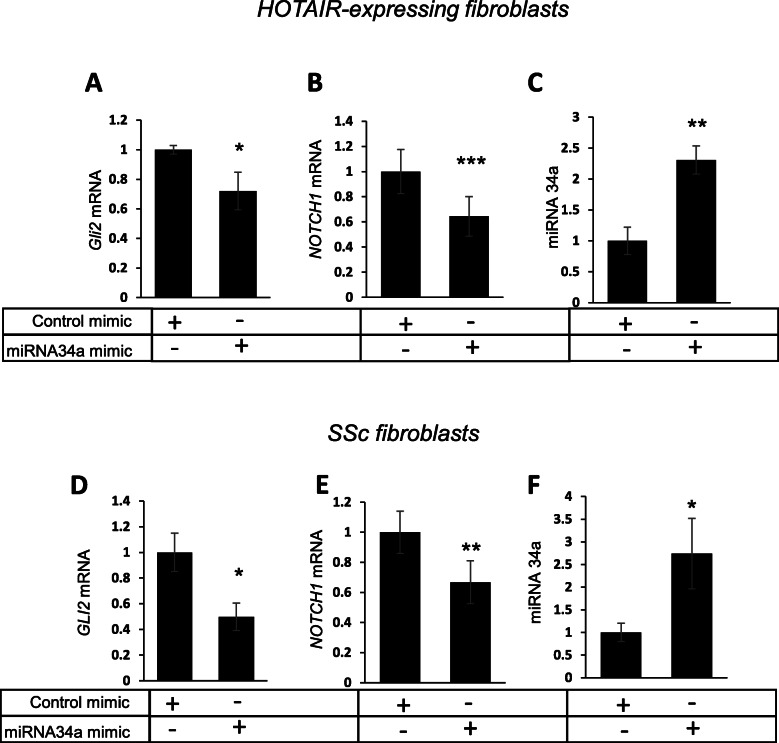
HOTAIR suppresses miRNA-34a to enhance Notch-mediated GLI2 expression. RNA was extracted from HOTAIR-expressing fibroblasts transfected with an miRNA-34a mimic or a scramble miRNA mimic. *GLI2* (**a**), *NOTCH1* (**b**) and miRNA-34a (**c**) transcript levels were analysed. Graphs represent the mean and standard error for three independent experiments. RNA was extracted from SSc fibroblasts transfected with miRNA-34a mimic or a scramble miRNA mimic. *GLI2* (**d**), *NOTCH1* (**e**) and miRNA34a (**f**) transcript levels were analysed. Graphs represent the mean and standard error for three independent experiments*.* *p < 0.05, **p < 0.01, ***p < 0.001

Previously, we have shown that HOTAIR enhances NOTCH1 expression through the suppression of miRNA-34a [11]. Therefore, we hypothesised that HOTAIR’s ability to suppress miRNA-34a might be important for GLI2 expression. To test this hypothesis, we transfected a miRNA-34a mimic or a scrambled control miRNA in HOTAIR-expressing fibroblasts. Rescue of miRNA-34a expression reduced *GLI2* and *NOTCH1* mRNA levels in HOTAIR-expressing fibroblasts (Fig. 2a, b). Transfection of the miRNA-34a mimic in SSc fibroblasts also led to a significant reduction in *GLI2* expression levels (Fig. 2d). Mechanistically, miRNA-34a repressed expression of *NOTCH1* transcripts in SSc fibroblasts (Fig. 2e). Of note, the miRNA-34a mimic transfection resulted in 2–2.5-fold increase in expression over basal both in HOTAIR- and SSc fibroblasts (Fig. 2c, f), suggesting that small changes in miRNA-34a are sufficient to downregulate *NOTCH1* and *GLI2* expression. Taken together, these data indicate that HOTAIR stimulates *GLI2* expression through downregulation of miRNA-34a, which controls *NOTCH1*.

### HOTAIR drives GLI2 expression through epigenetic changes directed by EZH2

**Fig. 3:**
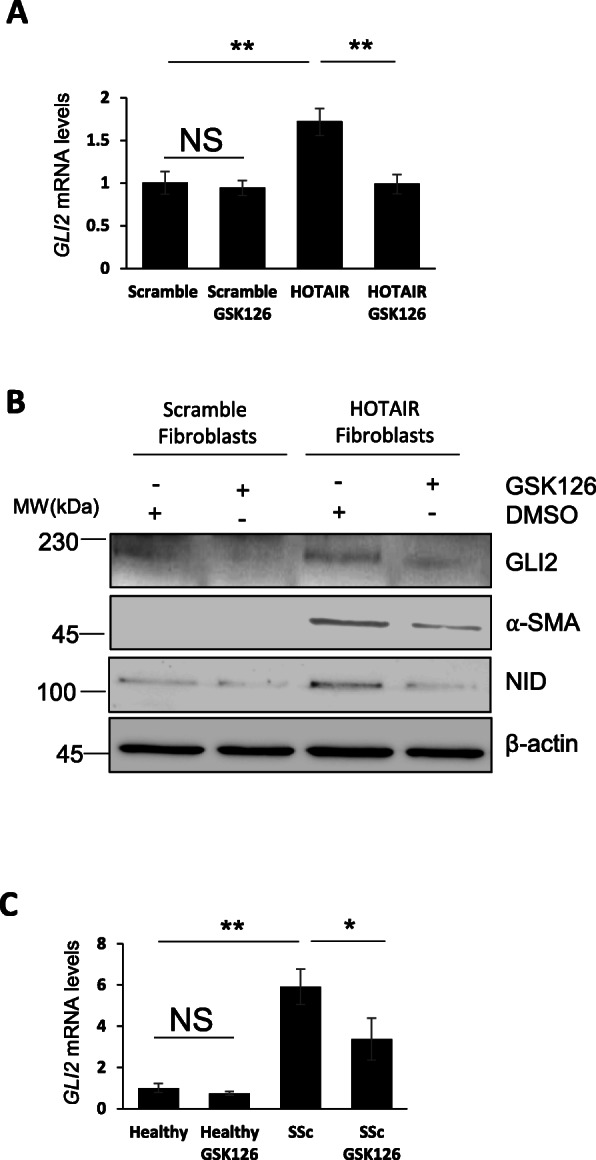
HOTAIR drives GLI2 transcription through EZH2 in HOTAIR-expressing and SSc fibroblasts. RNA and protein were extracted from scramble and HOTAIR-expressing fibroblasts. In addition, the scramble and HOTAIR-expressing fibroblasts were treated with the EZH2 inhibitor GSK126. **a** *GLI2* transcript levels were analysed. Graph represents the mean and standard error for three independent experiments. **b** GLI2, α-SMA and NID protein levels were assessed by western blot. β-actin was used as a loading control. **c** RNA was extracted from healthy and SSc fibroblasts. In addition, healthy and SSc fibroblasts were treated with the EZH2 inhibitors GSK126. *GLI2* transcript levels were analysed for both inhibitors. Graphs represent the mean and standard error for three independent experiments*.* *p < 0.05, **p < 0.01, ***p < 0.001

One of the best known roles of lncRNA HOTAIR is the activation of the polycomb repressor complex (PRC2) [25]. We therefore investigated whether inhibition of the PRC2 enzyme EZH2 in HOTAIR-expressing fibroblasts affected the expression of GLI2. Treatment of HOTAIR-expressing fibroblasts with the EZH2 inhibitor GSK126 for 48 h strongly reduced *GLI2* transcript levels, but the inhibitor had no effect on *GLI2* levels in the scramble fibroblasts (Fig. 3a). Consistent with these findings, we observed a strong reduction in GLI2 protein (70% reduction in the GSK126-treated fibroblasts) in the HOTAIR-expressing fibroblasts treated with the inhibitor (Fig. 3b). We also observed a reduction in α-SMA and NID levels in the HOTAIR-expressing fibroblasts treated with GSK126 (Fig. 3b), as previously described [11]. Inhibition of EZH2 in SSc patient-derived fibroblasts produced similar results. In SSc fibroblasts, GSK126 treatment reduced *GLI2* mRNA levels by 45% (*p* < 0.05) (Fig. 3c). Interestingly, GSK503, a second EZH2 inhibitor that is structurally similar to GSK126, showed similar results, suppressing *GLI2* mRNA to levels comparable to dermal fibroblasts derived from healthy control skin biopsies (data not shown).

### Overexpression of HOTAIR leads to aberrant canonical Hedgehog signalling

**Fig. 4:**
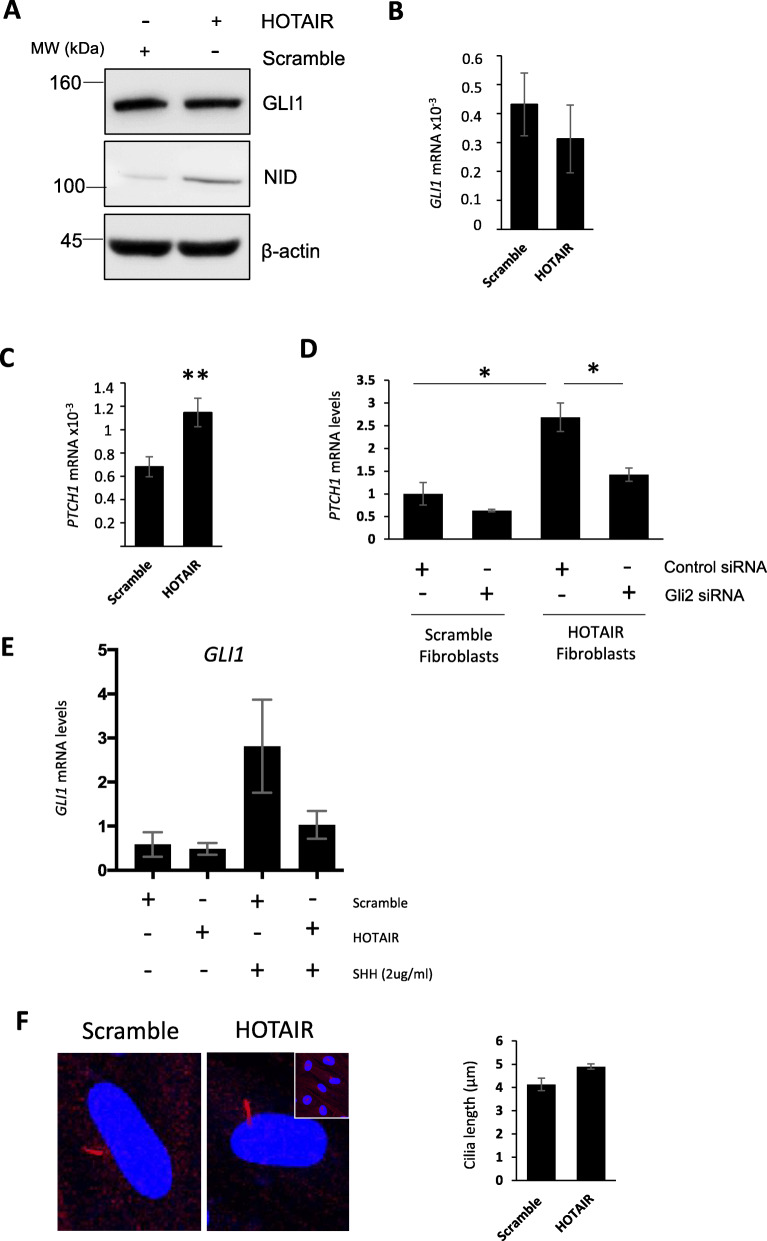
Overexpression of HOTAIR does not induce GLI1 expression and prevents SHH-mediated induction of GLI1. Dermal fibroblasts were transduced with lentiviruses containing a scramble or HOTAIR sequence. RNA and protein were extracted from these cells. **a** GLI1 and NID protein levels were analysed by western blot. β-actin was used as a loading control. *GLI1* (**b**) and *PTCH1* (**c**) transcript levels were analysed. Graphs represent the mean and standard error for three independent experiments. RNA was extracted from scramble and HOTAIR-expressing fibroblasts. In addition, the scramble and HOTAIR-expressing fibroblasts were transfected with GLI2 siRNA. A scramble control siRNA was transfected as control. *PTCH1* (**d**) transcript levels were assessed. Graphs represent the mean and standard error for three independent experiments. **e** RNA was extracted from serum depleted scramble and HOTAIR-expressing fibroblasts. In addition scramble and HOTAIR-expressing fibroblasts were stimulated with SHH ligand for 24 h. *GLI1* transcript levels were analysed. Graph represents the mean and standard error for three independent experiments. **f** Scramble and HOTAIR-expressing fibroblasts were stained for an antibody specific for acetylated α-tubulin and visualised with an Alexa 594-conjugated secondary antibody. DAPI was used to visualise the nuclei (blue). The images are individual cells at a magnification of × 63 with a wider field of view in the insert. Graph represents mean cilium lengths from 4 independent experiments where 30 cells were measured*.* *p < 0.05, **p < 0.01, ***p < 0.001

GLI2 is the main transcriptional activator of the Hh pathway. In the absence of Hh ligand stimulation, GLI2 exists in two forms: a full-length low activity activator and a shorter processed transcriptional repressor. Upon binding of a Hh ligand to PTCH1, processing is inhibited and full-length GLI2 is phosphorylated at the primary cilium to maximally increase its transcriptional activity [21]. GLI2 activation leads to *GLI1* induction at the transcriptional level, as well as upregulation of other GLI target genes, such as *PTCH1.* To determine whether GLI2 upregulation by HOTAIR resulted in activation of the canonical Hh pathway, we quantified the expression of *GLI1* and *PTCH1*, the hallmarks of GLI-dependent transcription. As shown in Fig. 4a and b, expression of GLI1 at protein and transcript levels was similar in scramble and HOTAIR-expressing fibroblasts. However, we observed a 1.6-fold upregulation of *PTCH1* mRNA levels (Fig. 4c) that was prevented by silencing of GLI2 expression (Fig. 4d). Next, we evaluated the effect of modulators of the EZH2/Notch/miRNA-34a pathway on GLI1 levels. Consistent with the lack of GLI1 induction by upregulation of GLI2, we observed no effects of EZH2 and gamma secretase inhibitors and miRNA-34a rescue on GLI1 expression in HOTAIR-expressing fibroblasts (Fig. S2A) or in SSc fibroblasts (Fig. S2B).

Given that higher levels of GLI2 might confer a heightened sensitivity of HOTAIR-expressing fibroblasts to Hh ligands, we investigated their responsiveness to SHH. We stimulated scramble and HOTAIR-expressing fibroblasts with 2 μg/ml SHH for 24 h. Unexpectedly, while scramble cells showed a 6-fold increase in *GLI1* expression, HOTAIR-expressing fibroblasts exhibited a weak response to SHH of about 2-fold (Fig. 4e). We suspected that HOTAIR expression could be altering primary cilia, essential for canonical SHH signalling [26]. However, HOTAIR did not alter the cilium length or gross structure, as shown with acetylated α-tubulin staining (Fig. 4f).

### GLI2 is essential for HOTAIR-mediated myofibroblastic transformation

**Fig. 5:**
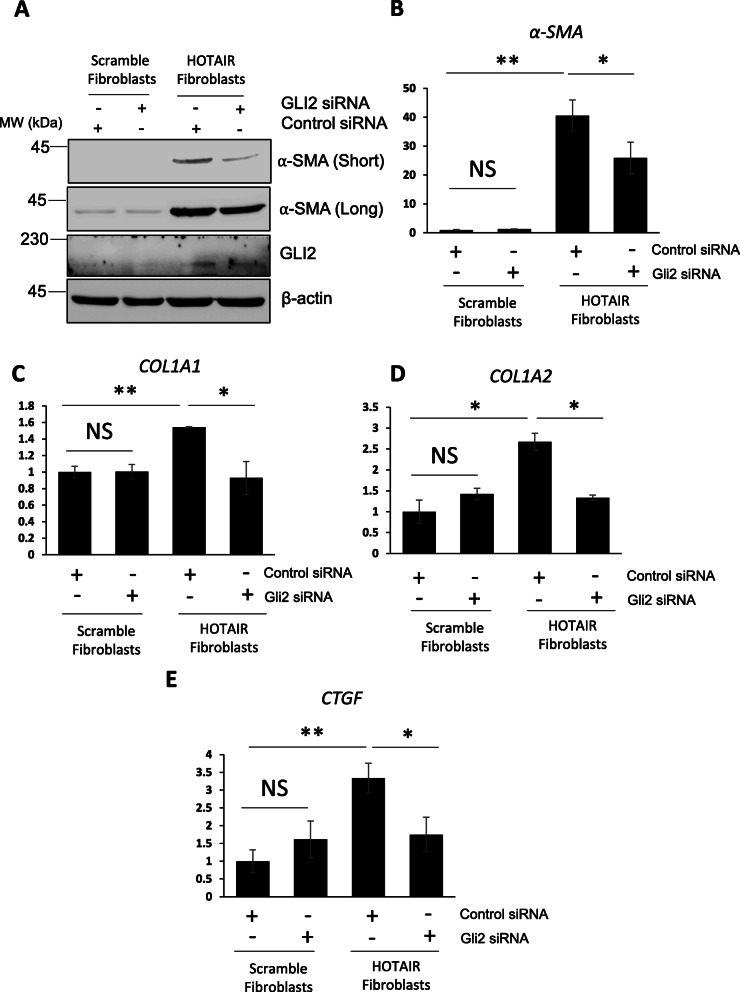
GLI2 silencing leads to a reduction in HOTAIR-mediated fibroblast activation. RNA and protein were extracted from scramble and HOTAIR-expressing fibroblasts. In addition, the scramble and HOTAIR-expressing fibroblasts were transfected with siRNA specific for GLI2. A scramble control siRNA was transfected into the other cell conditions. **a** α-SMA and GLI2 protein levels were assessed by western blot. β-actin was used as a loading control. *α-SMA* (**b**), *COL1A1* (**c**), *COL1A2* (**d**) and *CTGF* (**e**) transcript levels were assessed by qPCR. Graphs represent the mean and standard error for three independent experiments*.* *p < 0.05, **p < 0.01, ***p < 0.001

**Fig. 6:**
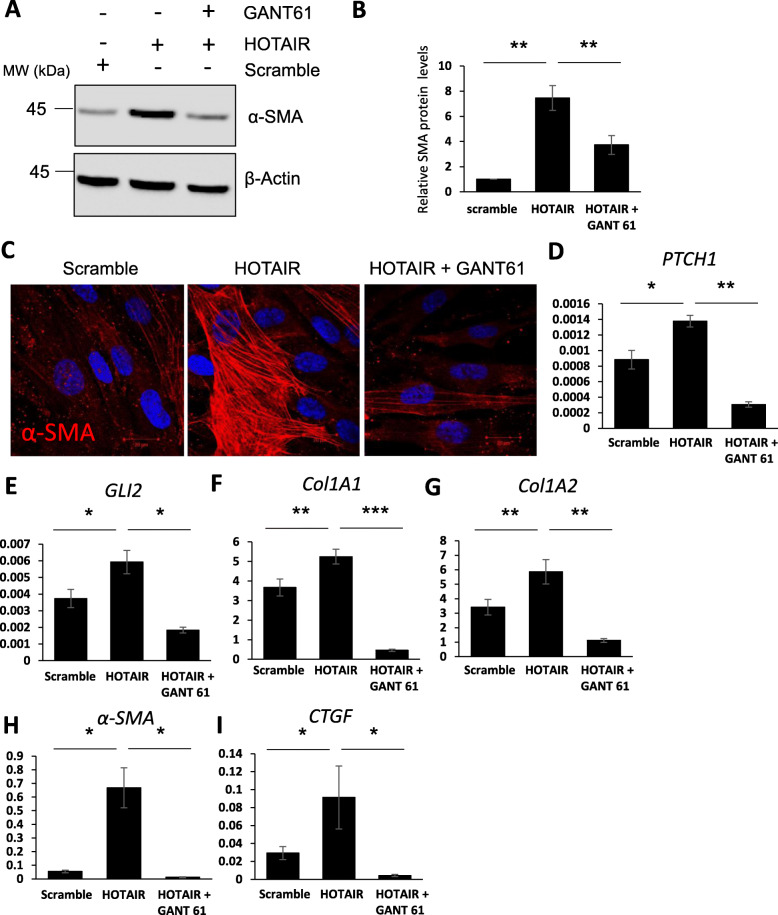
Inhibition of GLI2 leads to a reduction in HOTAIR-mediated fibroblast activation. RNA and protein were extracted from scramble and HOTAIR-expressing fibroblasts. In addition, HOTAIR-expressing fibroblasts were treated with the GLI inhibitor GANT61. **a** α-SMA protein levels were assessed by western blot. β-actin was used as a loading control. **b** Graph represents mean and standard error for densitometry analysis of the α-SMA western blots. **c** Scramble and HOTAIR-expressing fibroblasts were stained with an antibody specific for α-SMA. In addition, HOTAIR-expressing fibroblasts were treated with the GANT61. The α-SMA antibody was visualised with a mouse-specific Alexa 594-conjugated secondary antibody. DAPI was used to visualise the nuclei (blue). Red scale bars represent 20 μm. *PTCH1* (**d**), *GLI2* (**e**), *COL1A1*(**f**), *COL1A2* (**g**), *α-SMA* (**h**) and *CTGF* (**i**) transcript levels were analysed. Graphs represent the mean and standard error for three independent experiments*.* *p < 0.05, **p < 0.01, ***p < 0.001

To determine whether the increase in GLI2 expression observed in HOTAIR-expressing cells is a mediator of pro-fibrotic activation, we silenced GLI2 in the HOTAIR-expressing fibroblasts with siRNA and assessed pro-fibrotic marker expression. Reduction of *GLI2* mRNA levels in HOTAIR-expressing fibroblasts by 80% reduced the expression of the pro-fibrotic markers *α-SMA*, *COL1A1*, *COL1A2* and *CTGF* (Supp. Figure 1B and Fig. 5a–e). Depletion of GLI2 in the scramble control fibroblasts had no effect on pro-fibrotic marker expression. This suggests that GLI2 is an essential mediator of fibroblast activation by HOTAIR. In a complementary approach, we treated HOTAIR-expressing fibroblasts with the dual GLI1/GLI2 inhibitor GANT61 for 48 h. GANT61 reduced α-SMA protein levels in HOTAIR-expressing fibroblasts (Fig. 6a–c). GANT61 treatment decreased *PTCH1* and *GLI2* levels (Fig. 6d, e), suggesting the inhibitor was functional. In addition, we observed a reduction in mRNA levels of *COL1A1*, *COL1A2*, *α-SMA* and *CTGF* transcript levels in HOTAIR-expressing fibroblasts treated with the GLI inhibitor (Fig. 6f–i).

**Fig. 7:**
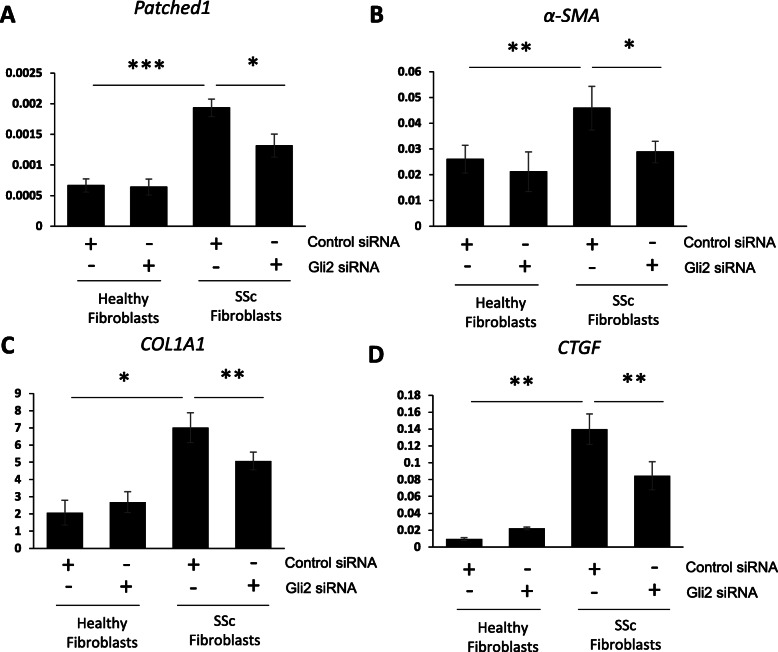
Depletion of GLI2 leads to reduced pro-fibrotic marker expression in SSc fibroblasts. RNA were extracted from healthy and SSc fibroblasts. In addition, the healthy and SSc fibroblasts were transfected with siRNA specific for GLI2. A scramble control siRNA was transfected into the other cell conditions. *PTCH1* (**a**) *α-SMA* (**b**), *COL1A1* (**c**) and *CTGF* (**d**) transcript levels were assessed by qPCR. Graphs represent the mean and standard error for three independent experiments*.* *p < 0.05, **p < 0.01, ***p < 0.001

**Fig. 8:**
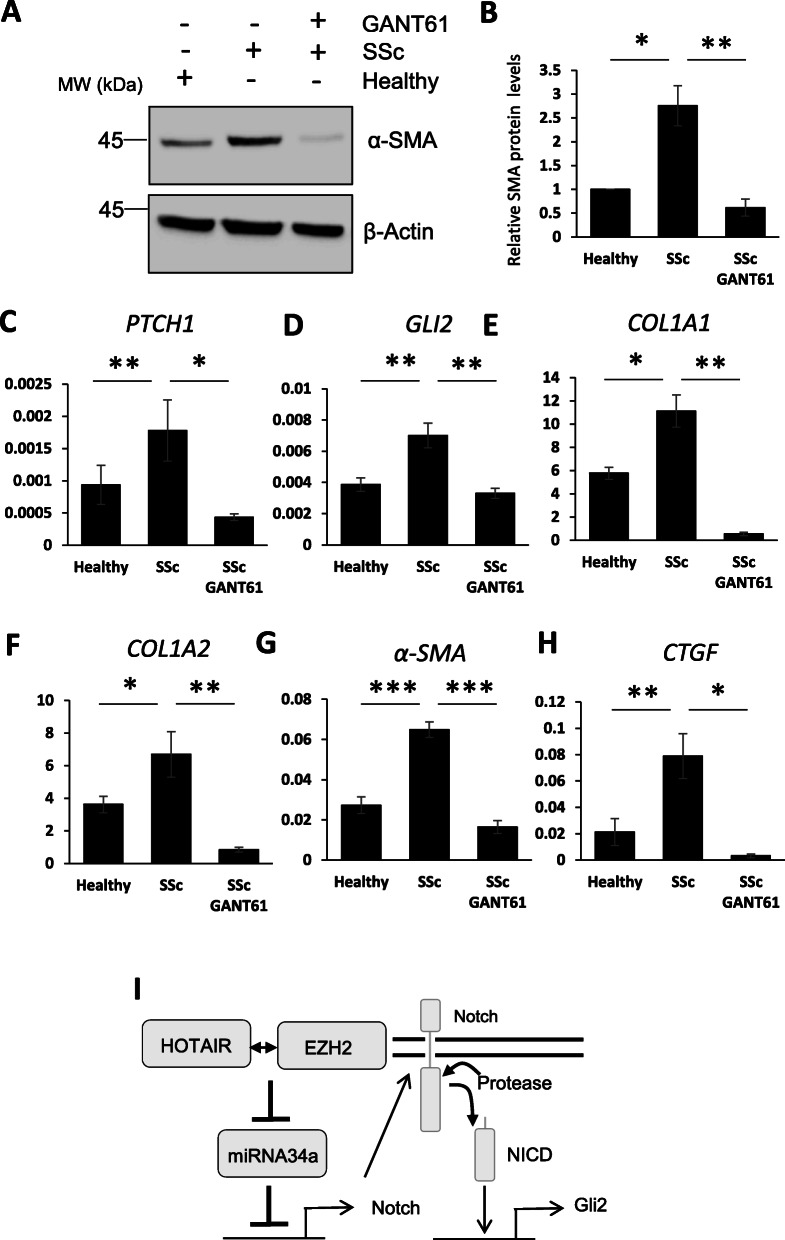
GLI2 inhibition reduces pro-fibrotic markers in SSc fibroblasts. RNA and protein were extracted from healthy and SSc fibroblasts. In addition, SSc fibroblasts were treated with the Gli inhibitor GANT61. **a** α-SMA protein levels were assessed by western blot. β-actin was used as a loading control. **b** Graph represents the mean and standard error for densitometry analysis of the α-SMA western blots. *PTCH1* (**c**), *GLI2* (**d**), *COL1A1* (**e**), *COL1A2* (**f**), *α-SMA* (**g**) and *CTGF* (**h**) transcript levels were analysed. Graphs represent the mean and standard error for three independent experiments. **i** Schematic of the HOTAIR/EZH2/miRNA-34a/Notch-mediated activation of GLI2. *p < 0.05, **p < 0.01, ***p < 0.001

Downregulation of GLI2 by siRNA depletion or inhibition with GANT61 reduced fibrotic marker expression levels in SSc fibroblasts (Figs. 7a-d and 8 a-h), as previously described [27]. Taken together, this data suggests that HOTAIR ability to regulate GLI2 expression is important for its capacity to induce pro-fibrotic activation (Fig. 8l).

## Discussion

SHH expression is upregulated in SSc skin and serum [28], with increased expression of PTCH1 and PTCH2 and the transcription factors GLI1 and GLI2 [5, 6]. It is noteworthy, though, that unlike GLI1, GLI2 is not a transcriptional target of the Hh pathway and its overexpression in SSc is putatively attributed to the known TGF-β/Smad-induced activation of the *GLI2* promoter [27]. Activation of Hh signalling in fibroblasts was shown to upregulate markers of myofibroblast activation such as COL1A1, COL2A1 and α-SMA and increased stress fibre formation [29]. The data in the literature shows that SSc fibroblasts maintain high levels of GLI2 expression in vitro. This suggests that epigenetic factors play an important role in this observation. In this study, we show that the PRC2 complex in cooperation with the lncRNA HOTAIR drives GLI2 expression in vitro through their ability to enhance Notch signalling.

The data presented here provides further mechanistic insight into the ability of HOTAIR to drive the activation of dermal fibroblasts. Here we show that HOTAIR drives pro-fibrotic markers in dermal fibroblasts partly through increased expression of the pro-fibrotic GLI2 protein. This is mediated through the suppression of the miRNA-34a which releases NOTCH1 suppression and leads to GLI2 transcription. Questions remain as to whether NID (in cooperation with the transcription factor CSL) directly binds to the GLI2 promoter and initiates transcription or there are further intermediates in the pathway. Additionally, it is not clear whether GLI2 directly initiates the transcription of the pro-fibrotic markers in SSc fibroblasts. These are interesting questions that need to be addressed in further studies.

Transcription of GLI2 has been shown to be driven by TGF-β in SSc [28]. Therefore, HOTAIR/Notch may cooperate with TGF-β to enhance GLI2 expression. There is evidence of cooperation between Notch and TGF-β signalling. Notch signalling is linked to phospho-SMAD activity [30] and TGF-β stimulation is known to enhance Hes1 expression through the interaction between Notch intracellular domain and SMAD3 [31]. In SSc fibroblasts, HOTAIR may prime the fibroblasts by enhancing Notch expression and this allows TGF-β to enhance GLI2 expression.

One caveat of this study is HOTAIR enhances GLI2 expression but not GLI1. This is interesting as GLI1 is a major transcriptional target of GLI2 in the Hh pathway [21]. Hh signalling primarily initiates at the cilia on the cell surface. Therefore, it is possible HOTAIR alters the cilium function in the fibroblasts, therefore altering the way the fibroblasts respond to SHH. We showed that HOTAIR does not alter the length of cilia (Fig. 4f) but it may alter their composition or ultrastructure, which may prevent the ability of the HOTAIR-expressing fibroblasts to respond to SHH.

Finally, this study has implications beyond fibrotic conditions. The Hh pathway is known to be overexpressed and deregulated in a number of cancers [32]. There is a new body of evidence that HOTAIR is also overexpressed in a number of cancers [33, 34], which suggests that HOTAIR may play a role in SHH dysregulation in cancer. Altogether, the effect of HOTAIR expression on GLI2 gives an important mechanistic insight on the profound epigenetic changes driven by PRC2 during fibrosis, which would be amenable of therapeutic targeting.

## Conclusion

We have identified a novel mechanism in which the Hh-responsive transcription factor GLI2 is upregulated in SSc fibroblasts. The long non-coding RNA HOTAIR increases GLI2 expression through enhanced Notch signalling. This provides a greater understanding of HOTAIR-mediated fibrosis in systemic sclerosis.

## Supplementary Information

**Additional file 1: FigureS1.** Inhibition of GLI2 does not affect NOTCH1 expression. RNA was extracted from scramble and HOTAIR-expressing fibroblasts. In addition, scramble and HOTAIR expressing fibroblasts were transfected with siRNA specific for GLI2. A scramble control siRNA was transfected into the other cell conditions. *NOTCH1* (A) and *GLI2* (B) transcript levels were analysed. Graphs represent the mean and standard error for three independent experiments. RNA was extracted from scramble and HOTAIR-expressing fibroblasts. In addition, HOTAIR-expressing fibroblasts were treated with the GLI inhibitor GANT61. (C) *NOTCH1* transcript levels were analysed. Graphs represent the mean and standard error for three independent experiments. RNA was extracted from healthy and SSc fibroblasts. In addition, healthy and SSc fibroblasts were transfected with siRNA specific for GLI2. A scramble control siRNA was transfected into the other cell conditions. *NOTCH1* (D) and *GLI2* (E) transcript levels were analysed. Graphs represent the mean and standard error for three independent experiments. *p < 0.05, **p < 0.01, ***p < 0.001. **Fig. S2.** GLI1 expression in HOTAIR and SSc fibroblasts is insensitive to EZH2 and gamma secretase inhibitors. (A) RNA was extracted from HOTAIR expressing fibroblasts. In addition, HOTAIR expressing fibroblasts were treated with the EZH2 inhibitor GSK126, the gamma secretase inhibitor R04929097 and transfected with miRNA-34a mimic. *GLI1* transcript levels were analysed. Graph represents the mean and standard error for three independent experiments. (B) RNA was extracted from SSc fibroblasts. In addition SSc fibroblasts were treated with the EZH2 inhibitor GSK126, the gamma secretase inhibitor R04929097 and transfected with miRNA-34a mimic. *GLI1* transcript levels were analysed. Graph represents the mean and standard error for three independent experiments.

## Data Availability

All data generated or analysed during this study are included in this published article. The datasets during and/or analysed during the current study are available from the corresponding author on reasonable request.

## Abbreviations

SSc: Systemic sclerosis
EZH2: Enhancer of Zeste Homology 2
TGF-β: Transforming growth factor beta
PRC2: Polycomb repressor complex 2
NID: Notch intracellular domain
GVHD: Graft versus host disease
SMO: Smoothened
PTCH1: Patched 1
Hh: Hedgehog
SHH: Sonic hedgehog
α-SMA: Alpha smooth muscle actin
