# Supplementary material for: Long non-coding RNA HOTAIR induces GLI2 expression through Notch signalling in systemic sclerosis dermal fibroblasts
Source: Arthritis Res Ther. 2020 Dec 10;22:286. doi: 10.1186/s13075-020-02376-9 (PMC7726858; doi:10.1186/s13075-020-02376-9)

Supplementary Figure 1: Silencing of GLI2 does not affect NOTCH1 expression

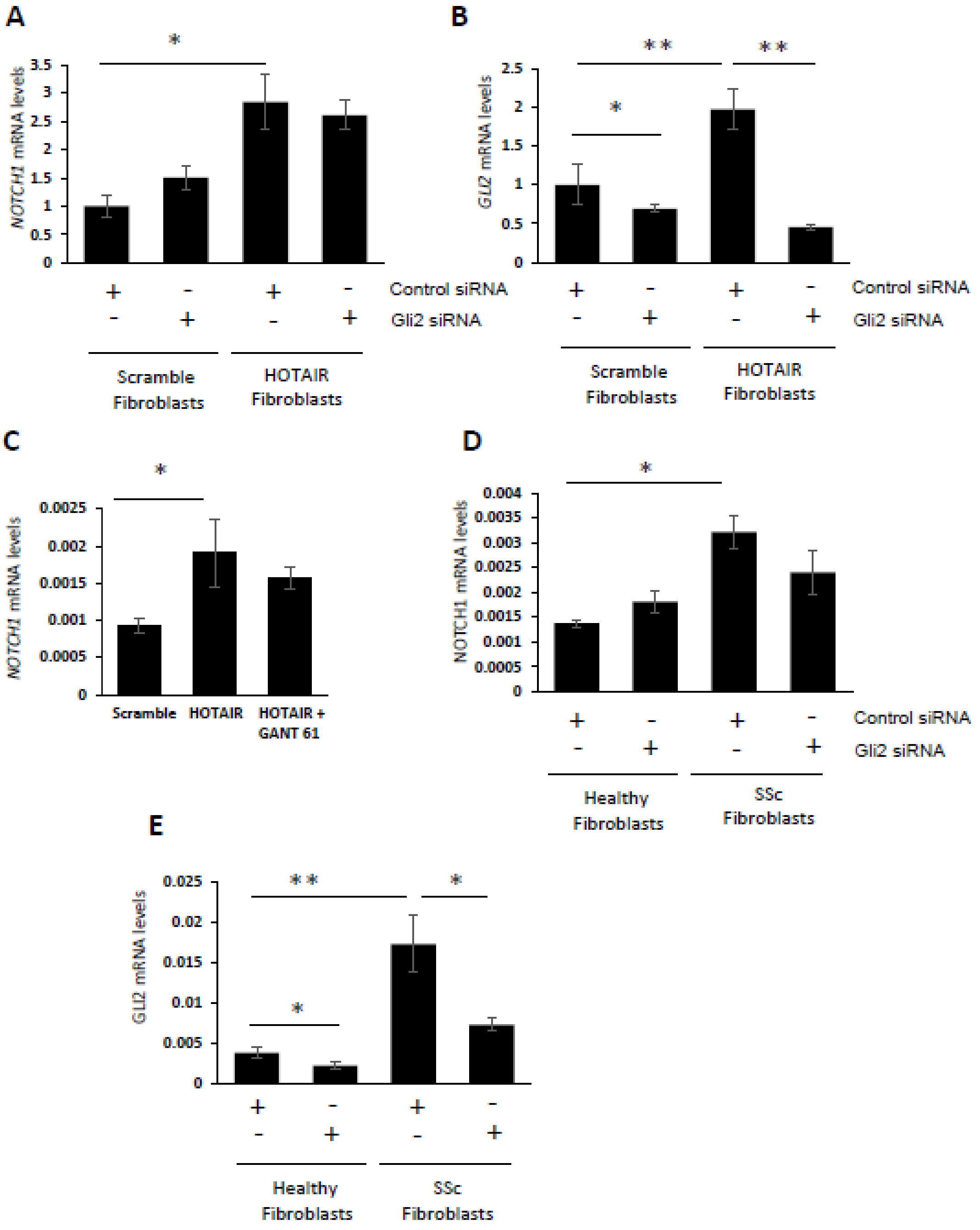

Supplementary Figure 2: GLI1 expression in HOTAIR and SSc fibroblasts is insensitive to EZH2 and gamma-secretase inhibitors

HOTAIR-expressing fibroblasts

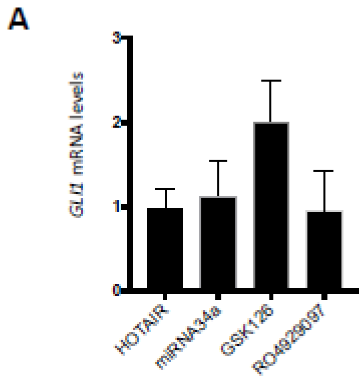

SSc fibroblasts

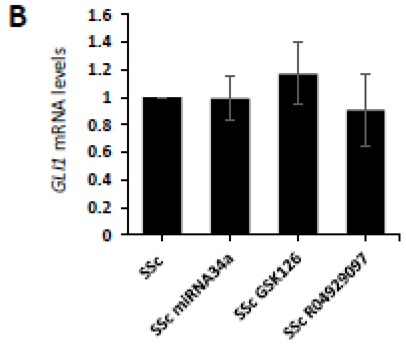

Supplement: Supplementary file 1 — Additional file 1: FigureS1. Inhibition of GLI2 does not affect NOTCH1 expression. RNA was extracted from scramble and HOTAIR-expressing fibroblasts. In addition, scramble and HOTAIR expressing fibroblasts were transfected with siRNA specific for GLI2. A scramble control siRNA was transfected into the other cell conditions. NOTCH1 (A) and GLI2 (B) transcript levels were analysed. Graphs represent the mean and standard error for three independent experiments. RNA was extracted from scramble and HOTAIR-expressing fibroblasts. In addition, HOTAIR-expressing fibroblasts were treated with the GLI inhibitor GANT61. (C) NOTCH1 transcript levels were analysed. Graphs represent the mean and standard error for three independent experiments. RNA was extracted from healthy and SSc fibroblasts. In addition, healthy and SSc fibroblasts were transfected with siRNA specific for GLI2. A scramble control siRNA was transfected into the other cell conditions. NOTCH1 (D) and GLI2 (E) transcript levels were analysed. Graphs represent the mean and standard error for three independent experiments. *p < 0.05, **p < 0.01, ***p < 0.001. Fig. S2. GLI1 expression in HOTAIR and SSc fibroblasts is insensitive to EZH2 and gamma secretase inhibitors. (A) RNA was extracted from HOTAIR expressing fibroblasts. In addition, HOTAIR expressing fibroblasts were treated with the EZH2 inhibitor GSK126, the gamma secretase inhibitor R04929097 and transfected with miRNA-34a mimic. GLI1 transcript levels were analysed. Graph represents the mean and standard error for three independent experiments. (B) RNA was extracted from SSc fibroblasts. In addition SSc fibroblasts were treated with the EZH2 inhibitor GSK126, the gamma secretase inhibitor R04929097 and transfected with miRNA-34a mimic. GLI1 transcript levels were analysed. Graph represents the mean and standard error for three independent experiments. [file 13075_2020_2376_MOESM1_ESM.pdf]
